# Holmium and Thulium Fiber Laser Safety in Endourological Practice: What Does the Clinician Need to Know?

**DOI:** 10.1007/s11934-023-01168-3

**Published:** 2023-05-31

**Authors:** Patrick Juliebø-Jones, Bhaskar K. Somani, Peder Gjengstø, Mathias Sørstrand Æsøy, Christian Beisland, Øyvind Ulvik

**Affiliations:** 1 Department of Urology, Haukeland University Hospital, Bergen, Norway; 2 Department of Clinical Medicine, University of Bergen, Bergen, Norway; 3 EAU YAU Urolithiasis Group, Arnhem, Netherlands; 4 Department of Urology, University Hospital Southampton, Southampton, UK

**Keywords:** Ureteroscopy, Urolithiasis, Laser, Injury, Safety

## Abstract

**Purpose of Review:**

To summarise the literature on laser safety during endourological practice.

**Recent Findings:**

Holmium and Thulium Fiber laser are the two main energy sources in the current clinical practice. The latter may have superior properties, but more clinical studies are needed to formally establish this. Laser injury to urothelium is more dependent on user experience rather than laser type. Smaller laser fibres allow for lower intra-renal temperature profiles. Operators should pay close attention to laser technique including maintaining the safety distance concept and only firing the laser when tip is clearly in view. When adjusting laser settings, pay close attention to resultant power given the associated heat changes. Prolonged periods of laser activation are to be avoided for the same reason. Outflow can be manipulated such as with access sheath to mitigate temperature and pressure changes. There is still limited evidence to support the mandate for compulsory use of eye protection wear during laser lithotripsy.

**Summary:**

Lasers are the gold standard energy source for stone lithotripsy. However, the safe clinical application of this technology requires an understanding of core principles as well as awareness of the safety and technical aspects that can help in protecting patient, surgeon and operating staff.

## Introduction

The use of laser (light amplification by stimulated emission of radiation) as an energy source for endoscopic intracorporeal stone lithotripsy is a cornerstone of minimally invasive interventions such as ureteroscopy (URS) and minimally invasive percutaneous nephrolithotomy (PCNL) [1–3]. It is also likely to be one of the key factors as to why, and in contrast to shockwave lithotripsy (SWL), the case volume of URS has risen markedly across the world [4, 5]. The frequent introduction of new modifications such as pulse modulation as well as newer laser platforms, e.g. thulium fibre laser (TFL), will likely see the continuation of this upward trajectory [6]. Furthermore, given that the incidence of stone disease is also increasing, the overall demand for surgical intervention will also increase in the future [7]. The trends also include a shift towards greater proportion of primary or ‘hot’ URS being performed during an acute inpatient episode [8]. Therefore, all urologists, regardless of subspecialist interest, have a requirement and obligation to maintain a certain level of knowledge and expertise in laser lithotripsy. While URS is commonly reported as a *safe and effective* procedure, complications do occur including adverse events, which are sometimes directly attributable to laser usage [9]. These can involve patients, surgeons and operating staff alike. Examples to consider include thermal injury to the ureter causing subsequent stricture and fibre fracture, which can both cause scope damage when it occurs within the working channel and skin burn outside the scope while being held by the surgeon [10]. User error is the commonest underlying cause rather than the device failure itself. It is therefore important that all users have an appreciation of what complications can occur with lasers during stone lithotripsy as well as an understanding of the key safety aspects. While use of laser lithotripsy receives wide coverage in European guidelines, discussion is generally focused on procedural outcomes and complications associated with the operation itself [11]. Specific guidance and practical considerations for clinical practice are usually lacking.

Our aim was therefore to provide urologists with an overview of key elements of laser safety during endoscopic stone surgery and steps that can be taken to avoid injury and mitigate adverse events.

## Materials and Methods

Comprehensive review was performed of literature relating to laser safety during endoscopic stone surgery. Bibliographic databases searched included PubMed, EMBASE, Web of Science, Cochrane Library and Google Scholar. Relevant information has been collated and reviewed by the authors to deliver a practical summary for clinicians. The following core areas were identified and discussed: laser machine choice, laser fibre properties, laser technique, laser settings, handling the laser fibre and operating staff safety including eye protection.

### Choice of Laser Machine

**Fig. 1:**
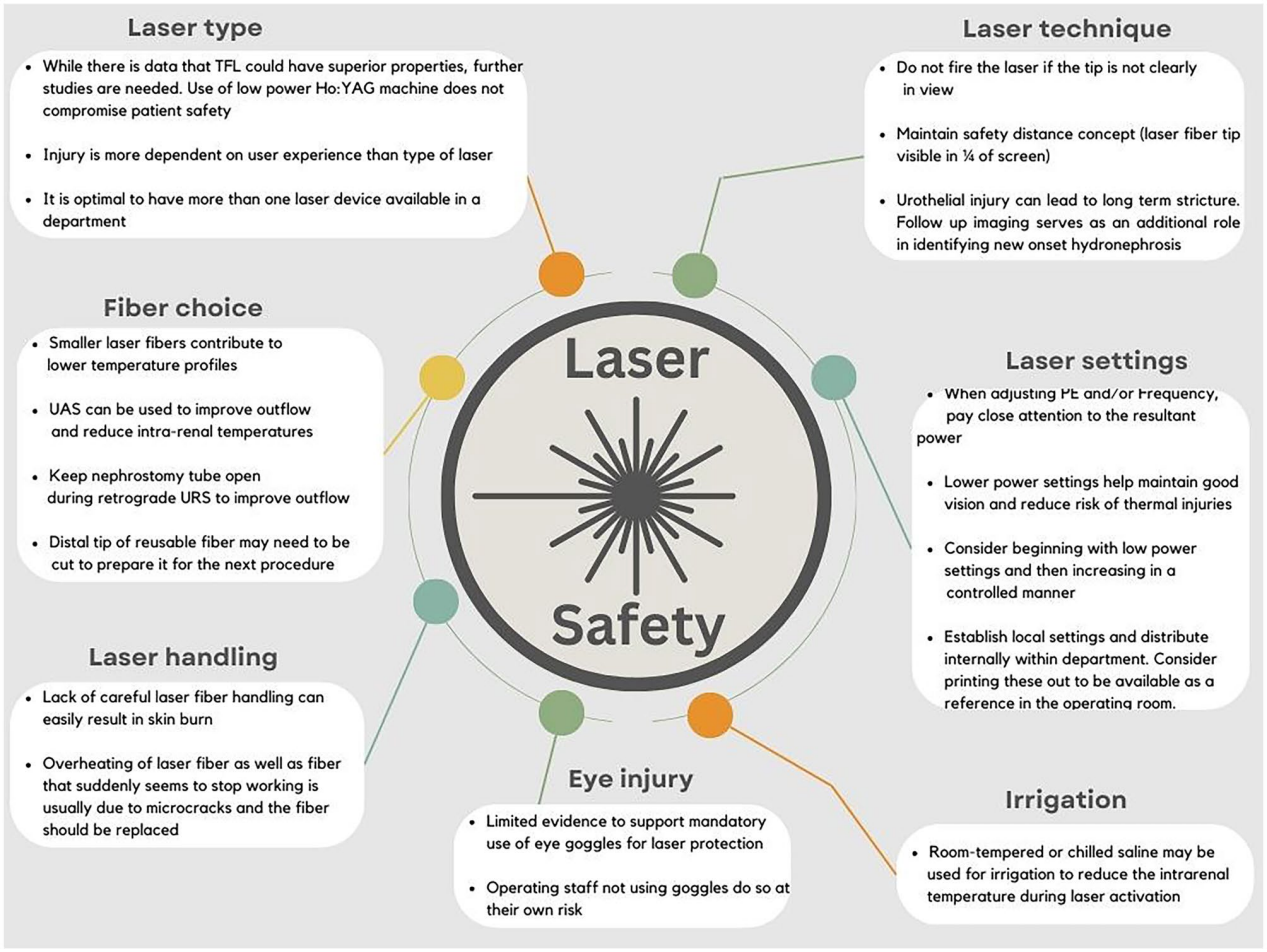
Summary of laser safety principles during surgery

Holmium:YAG (Ho:YAG) is the laser system most in current use worldwide for stone lithotripsy. TFL is a newer platform, which displays favourable characteristics such as a higher water absorption coefficient (WAC) and shorter aqueous optical penetration depth [6]. In clinical terms, this should translate to a lower ablation threshold and less risk of bleeding. With regard to intra-operative safety, a recent randomised clinical trial found TFL to result in significantly fewer events of bleeding impairing the operative view (5% vs. 22%, *p* = 0.014) (Table 1) [12]. However, the body of clinical data comparing TFL and Ho:YAG is still small, and further studies are needed to be able to discern if what we know from bench side studies actually results in a true safety benefit in the real intra-operative setting. Recent study by Sierra et al. found that regardless of laser type, injury to the urothelium is significantly higher when performed by a junior and less experienced urologist [13]. It seems therefore that the biggest determinant might be the technician and not the tool. Laser machine–related adverse events, e.g. hardware failure, are very rare but can occur [9]. Having more than one machine in the department is preferable for this reason (Fig. 1).

**Table 1:** Overview of key studies with results on laser safety

Author	Year	Study overview	Safety lessons
Laser type			
Ulvik et al. [2]	2022	Randomised clinical trial of Ho:YAG vs. TFL	• Significantly fewer intra-operative bleeding events impairing view associated with TFL compared to H.YAG (5% vs. 22%, *p* = 0.014)
Sierra et al. [13]	2022	Thermal injury associated with Ho:YAG vs. TFL (in vitro model)	• Higher risk of damage with higher power settings and less experienced surgeons
Laser fibre choice, handling and technique			
Paterson et al. [27]	2019	Survey of urologists in Endourological Society	• 19% of respondents had witnessed some kind of laser adverse event • Only 40% routinely wore laser protection eyewear • 76% had received formal laser training • 64% had formal laser safety policy at their hospital
Althunayan et al. [9]	2014	Review of US Manufacturer and User Facility Device Experience (MAUDE) database	• No eye injuries or deaths associated with Ho:YAG only skin burns to staff • Most adverse events due to fibre breakage
Tsaturyan et al. [24]	2022	Thermal effects of prolonged laser activation (in vitro model)	• Continuous activation at 12 Watts at 10 ml/min outflow caused threshold (43 °C) to be exceeded after only 1 min
Æsøy et al. [14•]	2022	Thermal effects of varying fibre size	• Larger fibres result in greater temperature changes
Ocular injury			
Villa et al. [29]	2016	Ocular injuries in ex vivo pig model with Ho:YAG	• Corneal damage occurs at 0–5 cm
Panthier et al. [31••]	2022	Ocular injuries in ex vivo pig model with TFL	• Corneal damage occurs at 0–5 cm

*TFL* thulium fibre laser

### Choice of Laser Fibre

While the choice between single use and reusable fibre may not be available to the operator, a size selection will usually be offered. Smaller laser fibres offer lower temperature profiles in the renal pelvis and in this regard can have a safety advantage [14•]. The reason is based on more irrigation flow down the fixed sized working channel. This can be compensated for by using a ureteral access sheath (UAS), but the user must then be vigilant to the relevant safety considerations when using this particular accessory device as improper use can lead to ureteric injuries and even evulsion [15]. The user must also take into account the size of scope relative to size of the UAS. Note also that in the study by Noureldin et al. using live pigs, the use of high-power Ho:YAG together with gravitational pressure could cause thermal injury even when large calibre UAS was used [16]. If the patient has a nephrostomy tube that is already in situ, this can be left open during the procedure, to ensure higher irrigational flow. As well as the outflow, the inflow can be adjusted to influence temperature. The use of room-tempered or even chilled irrigation fluid may be beneficial during continuous high-power laser activation to avoid dangerous rise in intra-renal temperature. Irrigation can be augmented by raising the bag height as well as with the use of dedicated devices such as hand or foot pump. However, raised intra-renal pressure can also lead to harm with risk of perirenal haematoma and sepsis. In this sense, intra-renal temperature and pressure are twinned, and the user has to balance the equilibrium between the two. The use of chilled irrigation has been trialled in live pig setting and was found to delay the impact of thermal injury without affecting core body temperature, but no human studies exist to date [17]. If a reusable laser fibre is used, the tip (distal 2–5 cm) may need to be cut, to remove the most fragile part of the laser from the current procedure, to prepare it for the next procedure, thereby minimising the risk of laser fibre break or fracture.

### Laser Technique

It is recommended to avoid direct contact between the laser and urothelium (minimum 1-mm distance) [2]. The latter is highly sensitive to injury and perforation can therefore occur. In the event of this, a stent should be placed at the end of the procedure, and a low threshold should be undertaken for early termination of the case. From a more long-term perspective, ureteral stricture can occur as a result of such thermal injury. Indeed, follow-up imaging of the upper urinary tract not only serves to determine stone-free status but also to identify new-onset hydronephrosis, which can suggest post URS stricture formation. In contrast to renal stones, it is the centre of ureteral stones where laser firing should be focused. Where urothelial injury is identified intra-operatively, it is beneficial to formally record the severity in the operation note using a classification system such as that previously described by Traxer and Thomas [18]. If it is difficult to maintain correct positioning during fragmentation due to patient’s respiratory movement, consider the use of apnoea to minimise risk of inadvertent ureteral injury. To this end, apnoea may be difficult to achieve if URS is performed in spinal anaesthesia or sedation. As a rule, the laser should not be fired if the view is suboptimal. When positioning the tip of the laser fibre, this should be kept at approximately ¼ distance of the screen. This is commonly referred to as the safety distance concept [19]. This helps reduce risk of inadvertent injury to the distal portion of the scope. This latter section is the most commonly damaged location of a scope and where some of the most expensive components are housed, e.g. chip of digital scope [20].

Dusting technique is usually applied for renal stones. Ideally, the laser fibre tip should be moved across the surface of the stone in a painting manner rather than burning holes centrally through the stone. Painting over the surface allows for production of fine dust rather than production of bigger fragments. It might be difficult to dust a stone completely, and most often, production of multiple small fragments will be the result. These can be reduced even further by application of pop-corning technique where the laser fibre is placed centrally in a calyx and the laser is activated continuously for several seconds with relatively high energy at high frequency. It is important that the fibre is not in contact with the mucosa during pop-corning to avoid injury.

Dusting ureteral calculi may be difficult due to the limited space surrounding the stone. A painting movement across the stone surface may not be feasible, and fragmentation can be a better alternative in these situations. However, as peak power is lower using TFL compared to Ho:YAG, fragmentation using TFL may imply a different technique where the stone is ‘cut’ into smaller pieces rather than fragmented.

### Laser Settings

Together with direct urothelial damage, high intra-renal temperatures also cause injury as a result of protein denaturation. This can lead to post operative stricture formation and, in some cases, intra-operative bleeding requiring termination of the procedure. Settings used among urologists vary widely as do the start-up settings (also termed pre-sets) recommended by different laser manufacturers [21, 22]. This can lead to confusion, especially among residents and those performing laser lithotripsy occasionally during emergency as a duty surgeon rather than on a regular basis. Two key parameters the surgeon can manipulate are pulse energy (PE, measured in joules (J)) and frequency (Fr, measured in hertz (Hz)). Power (Watts) is determined accordingly (PE × Hz = W). It is crucial therefore that the surgeon pays close attention to these values, especially the power, which is ultimately the main determinant of temperature rise [23]. In practical terms, *power* = *heat* and high values should be avoided due to risk of thermal injury. A general principle that can be followed is that values below 10 W and 20–30 W should be avoided in the ureter and renal pelvis, respectively. In the case of laser lithotripsy of a bladder stone, there is less risk when employing higher power settings given the augmented irrigation. Loss of vision due to a blizzard effect, injury to urothelium, contact bleeding and high-intra-renal temperature can all lead to injury. It is therefore safer to use low settings (e.g. 0.3–0.6 J and 10–30 Hz), especially with less experienced surgeons. Even if it is in a location where higher power settings can be used, it is not necessarily more efficient and can result in carbonisation of the stone.

An added safety advantage of low start-up settings is that they can be gradually increased in a controlled way. The surgeon thereby avoids the stop/start of continuously adjusting to allow the blizzard effect to settle and gain adequate vision. In this regard, *patience over power* is recommended*.* Even when using lower power, the surgeon should be mindful of any long period of continuous laser activation time without a pause. Furthermore, the pause should be more than just momentary. Tsaturyan et al. recorded in vitro temperatures with continuous laser activation at 12 W for 10 min [24]. Temperatures as high as 83 °C were recorded. While we currently lack studies to provide clinical recommendations on exactly how long continuous laser activation can be done, as well as how long the pause in between these should really be, the surgeon should maintain the principle of avoiding prolonged periods of laser activation to reduce the risk of thermal damage [14•]. Again, given what we know about the relationship between power and heat, we recommend that continuous time periods of pedal activation are inversely related to power.

Changing the laser settings will impact the stone disintegration. Using low energy and high frequency will typically produce a fine dust, while higher energy at low frequency will fragment the stone [25]. Pop-corning can be achieved using medium energy at high frequency. However, the laser pulse profile also influences stone disintegration. High peak power facilitates fragmentation and longer pulse width results in less retropulsion and finer dust particles. Some laser machines have the opportunity for pulse modulation so that the surgeon can tailor the stone treatment.

### Handling the Laser Fibre

Laser fibres are expensive and also highly fragile and therefore should be handled with care. As well as injury due to inadvertent firing while the laser tip is still within the scope, the sharp silica tip can damage the lining of the working channel quite easily. It should therefore be passed up the scope in a gentle manner, keeping the scope as straight as possible. The fibre can be held in a stable position inside the flexible endoscope by tightening the locking screw for the working channel. Care should be taken when fixing the fibre inside a semirigid scope using the locking lever at the end of the working channel as this might cut the fibre unintendedly.

There is little evidence to support either single use or reusable fibres as being more likely to damage the scope. However, if using the latter, these should be checked for any sign of damage or small cracks that can result in energy loss that can both decrease laser efficacy and increase risk of fibre fracture though energy loss with resultant scope damage. It has previously been reported that over 50% of scopes requiring repair were found to have damage to the working channel [26]. One should avoid the use of excess force when initially inserting the distal portion of laser in the scope. The commonest injury to occur to operating staff is a skin burn [9]. This is typically caused by fracturing while being manipulated. In a survey on Ho:YAG use in endourology, Paterson et al. reported that 19% of respondents had witnessed some kind of laser injury [27]. That study also found that a formal hospital policy for safe laser usage was only present in 64%, and only 76% had ever received formal laser safety training. The use of the laser aiming beam is based on individual preference, but one added safety advantage of their use can be a lack of feedback with faulty fibre if the aiming beam is lost. Fibres that overheat and/or stop working for no reason are usually attributable to a microfracture, and the safest step is to replace the laser fibre.

### Eye Protection

In a review of adverse events related to the use of Ho:YAG over 20 years, no ocular injuries were found [9]. This was emphasised in a recent best practice statement from the Canadian Urological Association who concluded that evidence to support mandatory eye protection for this purpose is not contemporary [28]. The authors of that report also performed a survey of eyewear usage and found that only 19% of surgeons use them routinely. Placing goggles over prescription glasses can lead the surgeon to experience poorer views and therefore impair the preciseness of their surgical technique. Villa et al. studied the distance/energy relationship to induce eye damage in porcine eyes. Regardless of time or settings, no corneal damage was sustained at a distance of 5 cm [29]. Two studies have replicated this study model recently with TFL. Lee et al. found corneal damage to occur at a distance of 10 cm when using settings of 1 J/10 Hz. Only at zero distance, i.e. contact, was deep layer injury incurred. Laser-specific eyewear offered complete protection whereas prescription glasses only provided partial protection [30]. Panthier et al. performed a similar study but concluded that corneal damage was induced up to 5 cm across a wide range of settings when exposed for 1 s [31••]. Of course, as well as a barrier to bodily fluids, eyewear also serves as protection against cataract formation, one of the stochastic effects of ionising radiation [32••].

## Limitations

It should be borne in mind that the evidence in this area is nearly exclusively limited to relatively low levels such as pre-clinical experimental studies as well as expert opinion. There is a need for human studies evaluating some of these questions. In-built temperature control as well as possibly automated feedback to adjust settings is anticipated to be introduced in the future [20]. For now, our practice must be guided based on the principles we have gained in a largely bench side setting.

## Conclusion

Lasers are the gold standard for stone lithotripsy. However, the safe clinical application of this technology requires a knowledge of certain principles as well as awareness of the safety and technical aspects that can help in protecting patient, surgeon and operating staff alike. Appropriate pre- and intra-operative techniques can minimise potential injury in all areas of laser lithotripsy.
